# Stretchable and Wearable Triboelectric Nanogenerator Based on Kinesio Tape for Self-Powered Human Motion Sensing

**DOI:** 10.3390/nano8090657

**Published:** 2018-08-24

**Authors:** Shutang Wang, Minghui He, Bingjuan Weng, Lihui Gan, Yingru Zhao, Ning Li, Yannan Xie

**Affiliations:** 1 College of Energy, Xiamen University, Xiamen 361000, Fujian, China; wangshutang@stu.xmu.edu.cn (S.W.); minghuihe@hotmail.com (M.H.); ganlihui@xmu.edu.cn (L.G.); yrzhao@xmu.edu.cn (Y.Z.); ningli@xmu.edu.cn (N.L.); 2 Huli Street Community Health Service Center of Huli District, Xiamen 361000, Fujian, China; bingjuanweng@hotmail.com; 3 Key Laboratory for Organic Electronics and Information Displays & Institute of Advanced Materials (IAM), Jiangsu National Synergetic Innovation Center for Advanced Materials (SICAM), Nanjing University of Posts & Telecommunications, Nanjing 210023, Jiangsu, China

**Keywords:** triboelectric nanogenerator, energy harvesting, self-power active sensor, flexible and wearable electronics, biomechanical sensing

## Abstract

Recently, wearable, self-powered, active human motion sensors have attracted a great deal of attention for biomechanics, physiology, kinesiology, and entertainment. Although some progress has been achieved, new types of stretchable and wearable devices are urgently required to promote the practical application. In this article, targeted at self-powered active human motion sensing, a stretchable, flexible, and wearable triboelectric nanogenerator based on kinesio tapes (KT-TENG) haven been designed and investigated systematically. The device can effectively work during stretching or bending. Both the short-circuit transferred charge and open-circuit voltage exhibit an excellent linear relationship with the stretched displacements and bending angles, enabling its application as a wearable self-powered sensor for real-time human motion monitoring, like knee joint bending and human gestures. Moreover, the KT-TENG shows good stability and durability for long-term operation. Compared with the previous works, the KT-TENG without a macro-scale air gap inside, or stretchable triboelectric layers, possesses various advantages, such as simple fabrication, compact structure, superior flexibility and stability, excellent conformable contact with skin, and wide-range selection of triboelectric materials. This work provides a new prospect for a wearable, self-powered, active human motion sensor and has numerous potential applications in the fields of healthcare monitoring, human-machine interfacing, and prosthesis developing.

## 1. Introduction

Recently, wearable electronics have undergone a vast development and are highly desirable for commercial, medical, and military applications [1,2]. Among them, wearable human motion sensors have emerged as a promising sub-category and shown significant potential in biomedical monitoring, human-machine interfacing, sport sensing, and prosthesis development [3,4,5]. Conventional human motion monitoring technology mainly focuses on infrared-based photo-electric detection and force-induced changes in electrical parameters, such as resistance or capacitance [6,7,8]. However, those types of sensors normally necessitate external power-supply devices whose small power density and limited lifetime dramatically hinder the rapid advancement of this field [9,10]. To solve the above issues, self-powered sensing technology has been introduced and attracted significant interest as a crucial alternative [11]. The self-powered active sensor will generate electricity as a response to the external stimuli and the electric signal can reversely reflect the impact of the outside trigger [12]. Therefore, it is able to effectively and independently work without any external power sources and, hence, its accelerated progress is of great significance to the prevalence of wearable and flexible human motion sensors [13].

Originated from Maxwell’s displacement current, nanogenerators (NG) based on piezoelectric and triboelectric effects have been developing rapidly for efficiently harvesting environmental mechanical energy and self-powered active sensors [14,15,16,17,18]. The reported piezoelectric nanogenerators (PENGs) were mainly based on inorganic piezoelectric nanomaterials, such as ZnO and lead zirconate titanate [9]. Additionally, organic piezoelectric nanomaterials are promising for the application of PENG in flexible and wearable electronics due to their mechanical flexibility, easy processing, nontoxicity, and biocompatibility [19]. Recently, Khan et al. [20] demonstrated a self-powered strain sensor based on electrospun polyvinylidene fluoride (PVDF) nanofiber arrays to sensitively detect the fingers’ motion. Jeong et al. [21] reported a strong and flexible electronic vessel based on a functional PVDF fibrous membrane to monitor human motion and fluid pressure in real-time. Compared with PENG, triboelectric nanogenerators (TENGs) rely on a conjunction of triboelectrification and electrostatic induction, which has shown numerous advantages, including large output, high efficiency, low cost, simple fabrication, light weight, and being environmentally friendly [22,23,24,25]. Since the birth of TENG in 2012, extensive efforts have been devoted to this field and demonstrated its wide applications, ranging from self-charging systems and large-scale blue energy to self-powered active sensors [26,27,28,29,30]. For human motion sensors, TENGs have been reported to be able to act as self-powered devices for real-time monitoring [31,32,33,34,35]. Nevertheless, most of the previous works were based on the vertical contact-separation mode which requires a macro-scale air gap and the performance is dramatically determined by the separation distance [10]. The macro-scale air gap is generally unsuitable for wearable electronics because this type of structure cannot easily form conformal contact with skin and will consume extra space of the compact-structure wearable devices [10]. From this perspective, the devices based on the lateral mode without any gap inside may be applicable for wearable body motion sensors. Shi et al. [10] reported a self-powered flexible microfluidic sensor based on liquid-solid interface triboelectrification for pressure sensing and finger motion monitoring applications. However, the fabrication process was relatively complex and the stretchability of the device was out of consideration. Yi et al. [36] proposed a stretchable-rubber-based TENG with single-electrode mode which was used as a self-powered body motion senor. However, this structure requires at least one of the triboelectric layers to be stretchable, which largely restricts the broad range of material selections. Moreover, the issue of robustness and stability caused by the fatigue of the stretched materials may be a significant concern for this kind of device. Therefore, developing a new type of stretchable and wearable device with all the above crucial issues resolved is crucial for the practical application of TENG as a self-powered active human motion sensor.

In this work, we report a stretchable, flexible, and wearable kinesio-tape-based TENG (KT-TENG) relying on the lateral sliding working mode as a self-powered, active human motion sensor. The kinesio tapes are chosen due to their good biocompatibility, and excellent flexibility and durability [37,38]. By adopting the highly stretchable tapes as substrates and rationally designing the device structure, the triboelectric layers will not require being stretchable, which can greatly expand the range of the material selection and prolong the lifetime of the TENG. The KT-TENG is simply composed of two triboelectric layers (PET and Kapton films) with a back electrode and two kinesio tapes as substrates to, respectively, fasten opposite ends of two triboelectric layers. Once the KT-TENG is stretched, the two triboelectric layers will slide relatively in a plane, which will generate a potential difference between the two electrodes. The electric output signals of the KT-TENG are systematically investigated under different stretched displacements and bending angles. The results demonstrate that both the short-circuit transferred charge (*Q_SC_*) and open-circuit voltage (*V_OC_*) are linearly correlated to the displacements and bending angles. Furthermore, the KT-TENG is attached onto the human knee joint and human fingers to detect and monitor the movement of the knee and the human gestures, indicating its practical potential in self-powered human motion sensing. In comparison with the previously-reported devices, the KT-TENG, which does not require a macro-scale air gap inside, or triboelectric layers to be stretchable, exhibits numerous advantages including simple fabrication, compact structure without an air gap, superior stretchability and flexibility, excellent conformable contact with the skin, and wide-range selection of triboelectric materials. This work proposes a new device structure and approach for self-powered human motion sensor which may contribute profoundly to the further development of this field.

## 2. Materials and Methods

### 2.1. Materials of the KT-TENG

(1) The Kapton films, PET films, and kinesio tapes used in this work were commercial products purchased from DuPont (Shenzhen, China), Grafix (Ohio, the USA), and Decathlon (Shanghai, China), respectively. 

(2) The surface of the PET films was functionalized with nanostructures to enhance the triboelectric effect. Firstly, the PET films were washed with menthol, isopropyl alcohol, and deionized water, consecutively. Secondly, a thin layer of Au (~10 nm) was deposited onto the PET surface using direct-current (DC) magnetron sputtering, which can be used as “nanomasks” for the following etching process. Finally, inductively-coupled plasma (ICP) reactive ion etching was adopted to form the nanorod-like structures on the surface [39]. To be specific, Ar, O_2_, and CF_4_ gases were chosen as the reaction gases in the ICP chamber with flow ratio of 15.0, 10.0, and 30.0 sccm, respectively. One power source of 400 W was applied to produce a large density of plasma and the other power of 100 W was employed to accelerate the plasma ions. The PET film was etched for 40 s. The fabrication process has good repeatability. 

(3) Ag electrodes with a thickness of 100 nm are deposited onto the back sides of the Kapton and PET films through DC magnetron sputtering.

### 2.2. Fabrication of the KT-TENG

A piece of Kapton film was cut into a rectangle shape with the size of 1.5 × 5 cm^2^, and placed onto the top of a PET film with the size of 2 × 5 cm^2^. The overlap area is 1.5 × 4 cm^2^. The ends of the above triboelectric layers outside the overlap area were securely fixed to two kinesio tapes, respectively. The conducting copper wires were connected to the two Ag electrodes as the leads for electric output measurement. Finally, the whole device was packed and sealed with the two kinesio tapes.

### 2.3. Measurement of the KT-TENG

A programmable linear motor (LintMot E1100, LinMot, Spreitenbach, Switzerland) was applied to form reciprocating motions and stretch the KT-TENG with precise control of displacements. The *Q_SC_* and *V_OC_* were measured by Keithley 6514 system electrometer (Tektronix, Shanghai, China). Computer-controlled measurement software written in LabVIEW was employed to collect and record the experimental data.

## 3. Results and Discussion

**Figure 1:**
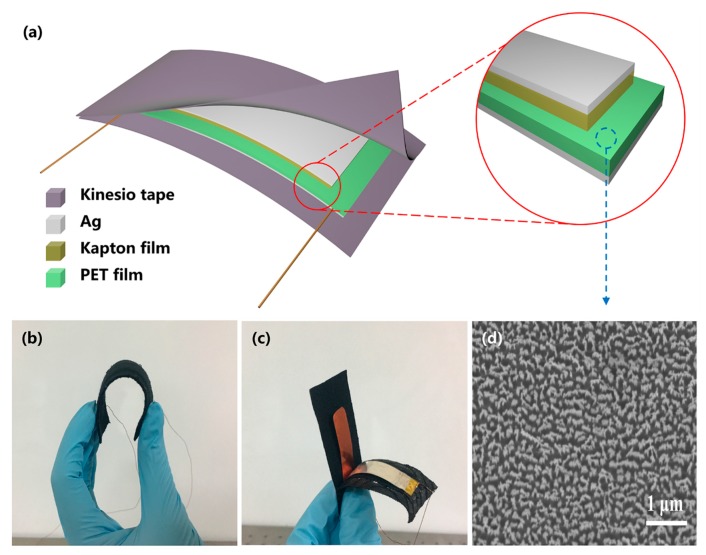
Device structure of the KT-TENG. (**a**) Schematic diagram of the KT-TENG. (**b**) A photograph showing the flexibility of the KT-TENG. (**c**) A photograph showing the inner structure of the KT-TENG. (**d**) SEM image of the nanorod-like structure on the PET surface.

Figure 1a show the device structure of the KT-TENG, which consists of a Kapton film on the top and a PET film at the bottom as the effective triboelectric layers. Both of the two films were coated with Ag back electrodes with a thickness of 100 nm through DC magnetron sputtering. The whole device was sealed and packed by two commercial kinesio tapes with high stretchability and good biocompatibility. The opposite ends of the two triboelectric layers were stuck to the respective kinesio tapes and, hence, the two films can slide relatively between each other during the whole device being stretched and released. Since all the materials used in the device fabrication are highly flexible, the whole devices exhibits excellent flexibility (as shown in Figure 1b) which is important to form conformal contact with the human body [10]. Figure 1c depicts the photograph of the KT-TENG showing the inner structure. As can be observed, the device is simple and compact in structure, which has potential value for large-scale fabrication. Furthermore, to enhance the surface roughness and, hence, the triboelectric effect [27,40], the PET film was functionalized with uniform nanorod-like structures on the surface [39], as can be seen from the scanning electron microscopy (SEM, Carl Zeiss AG, Oberkochen, Germany) image in Figure 1d. The detailed fabrication process for the KT-TENG is elaborated in the Materials and Methods Section.

**Figure 2:**
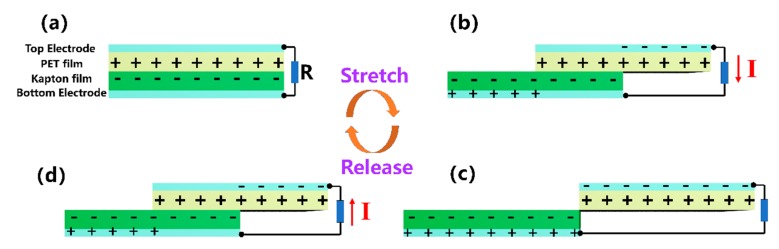
The working mechanism of the KT-TENG under the condition of being stretched. (**a**) The initial state. (**b**) the stretched state. (**c**) the maximum displacement of the stretched motion. (**d**) the released state.

The working mechanism of the KT-TENG relies on the coupling effect of triboelectrification and electrostatic induction, as schematically illustrated in Figure 2. Since the PET and the Kapton films have distinct abilities to attract electrons, electrons are injected from PET into Kapton when the two films are brought into contact, leaving the PET surface with positive charges and the Kapton surface with equal, but opposite charges. The relative polarity of materials can be explored from the triboelectric series diagram [22]. During the operation of the KT-TENG, the two triboelectric layers are set to contact closely and overlap with each other initially (Figure 2a). In this state, there is no potential difference between the two electrodes because of the electrostatic equilibrium. Once the device starts to be stretched, the PET film and the Kapton film make a relative lateral sliding outward (Figure 2b). This movement will induce the in-plane charge separation and, hence, a potential difference between the two electrodes owing to the decrease in the contact surface area. If the KT-TENG is connected with an external load, a transient current from the top electrode to the bottom electrode will be driven to neutralize the electric field induced by the triboelectric charges. This process will continue until the tribo-charged surfaces are entirely separated (Figure 2c). At this moment, another electrostatic equilibrium will be achieved. When the KT-TENG is released, the two triboelectric layers will slide inward and, again, contact with each other (Figure 2d). Correspondingly, another transient current will flow reversely from the bottom electrode to the top electrode until the two triboelectric layers return to the fully-aligned stacking position as shown in Figure 2a. Based on the above discussion, two alternating current peaks will be generated in the external circuit, with an equal number of charges flowing back and forth during one stretching-releasing cycle. 

**Figure 3:**
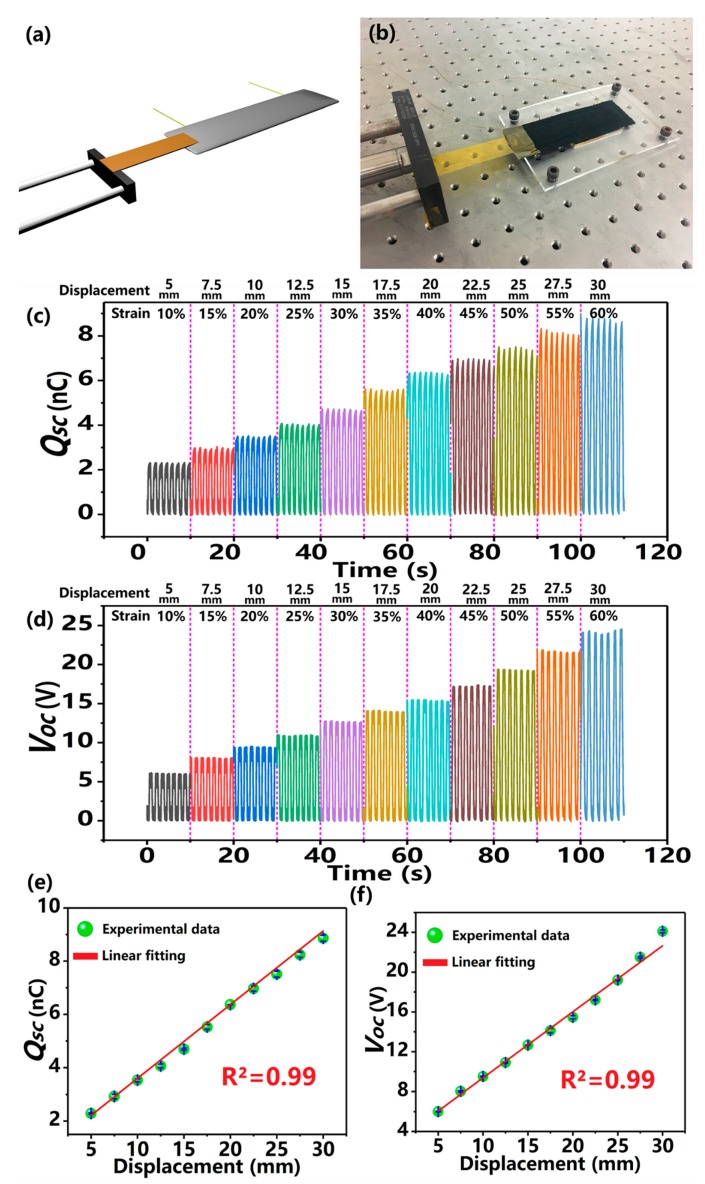
Dependence of the electric outputs on the stretched displacement. (**a**,**b**) Experimental setup of the electric signal measurement to stretch the KT-TENG. (**c**) The measured *Q_SC_* and (**d**) *V_OC_* of the KT-TENG under different stretched displacements. (**e**) The relationship between the *Q_SC_* and the stretched displacement. (**f**) The relationship between the *V_OC_* and the stretched displacement.

The experimental setup for electric output measurement of the KT-TENG is depicted in Figure 3a,b. One end of the KT-TENG is fastened to an acrylic sheet fixed on an optical table, with the other end connected to a programmable linear motor. The whole system is horizontally placed to ensure the horizontal stretching of the KT-TENG. The stretched displacement of the device can be precisely adjusted through the linear motor. The frequency of the stretched-released cycle is about 0.7 Hz. The strain is defined as the ratio of the stretched displacement and the initial length [36,41]. An electrometer is applied to record the electric signal in real-time. Based on the above apparatus, the relationship between the electric outputs (*Q_SC_* and *V_OC_*) and displacement can be systemically investigated. The *Q_SC_* and *V_OC_* of the KT-TENG with respect to different displacements from 5 mm to 30 mm (corresponding to different strains from 10% to 60%) are summarized in Figure 3c,d. It can be observed that the measured *Q_SC_* and *V_OC_* both increase with the stretched displacement. More specifically, when the KT-TENG is stretched with a small displacement of 5 mm corresponding to the strain of 10%, a voltage of 6.0 V and transferred charges of 2.3 nC are recorded. If the device is further stretched with displacements of 10, 20, and 30 mm (strains of 20%, 40%, and 60%), the *V_OC_* will be enhanced to and 9.5, 15.5, 24.1 V corresponding to the *Q_SC_* of 3.5, 6.4, and 8.9 nC. Figure 3e,f illustrate the plots of the peak values of the *Q_SC_* and *V_OC_* against the stretched displacements from 5 mm to 30 mm. The fitting of the data displaces a good linearity with adjusted R^2^ = 0.99 for both *Q_SC_* and *V_OC_*. The linear behavior of *Q_SC_* to displacements can be attributed to the linear correlation between *Q_SC_* and the separated surface area under the condition of uniform triboelectric charge distribution [42]. The *V_OC_* exhibits the similar variation tendency with *Q_SC_* according to the general relationship between the two key parameters in TENG [43]:

(1) $$V_{OC}=Q_{SC}/C_{TENG}$$

where *C_TENG_* is the capacitance of KT-TENG which is a constant. Therefore, both *Q_SC_* and *V_OC_* will increase linearly with the stretched displacements. Based on the above discussion, the KT-TENG can be applied as a self-powered active sensor for real-time displacement monitoring. It is worth noting that the maximum stretchability of the KT-TENG is 60% which is the stretched limit of the commercial kinesio tape we used in this work. If the KT-TENG is furtherly stretched in excess of 60%, the kinesio tape will be damaged and lose elasticity. 

**Figure 4:**
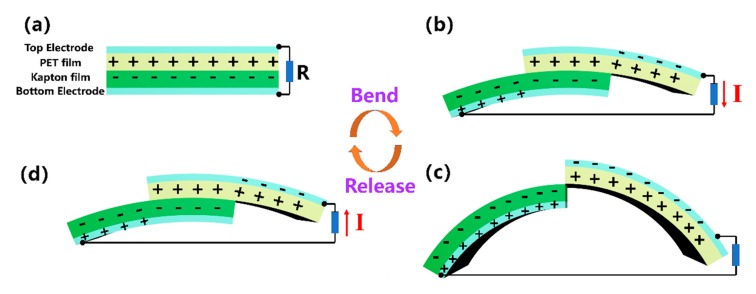
The working mechanism of the KT-TENG under the condition of being bent. (**a**) The initial state. (**b**) the bending state. (**c**) the maximum displacement of the bending motion. (**d**) the released state.

In consideration of the practical application for wearable sensor, the KT-TENG is required to work effectively under bending circumstances, such as bending motions of the elbow and knee joints. Since the device is highly flexible and stretchable, it is expected to achieve the above requirement. The possible working mechanism is schematically depicted in Figure 4, which is the same with that in Figure 2. Under the two circumstances, the electric energy is all generated by the relative sliding between the PET film and Kapton film (induced by the stretching motion for Figure 2 or bending motion for Figure 4). The initial state (as shown in Figure 4a) is similar to the in-plane stretching operation as discussed above. Since the TENG will be attached to the skin, the bending strain will stretch the device crookedly and initiate the two triboelectric layers to slide relatively, as illustrated in Figure 4b. Consequently, a potential difference between the two electrodes is generated to drive a current flow through the external circuit. This process will continue until another equilibrium state is achieved (Figure 4c). If the KT-TENG is released, the two triboelectric layers will slide back to the original state, resulting in a reverse current flow (Figure 4d). 

**Figure 5:**
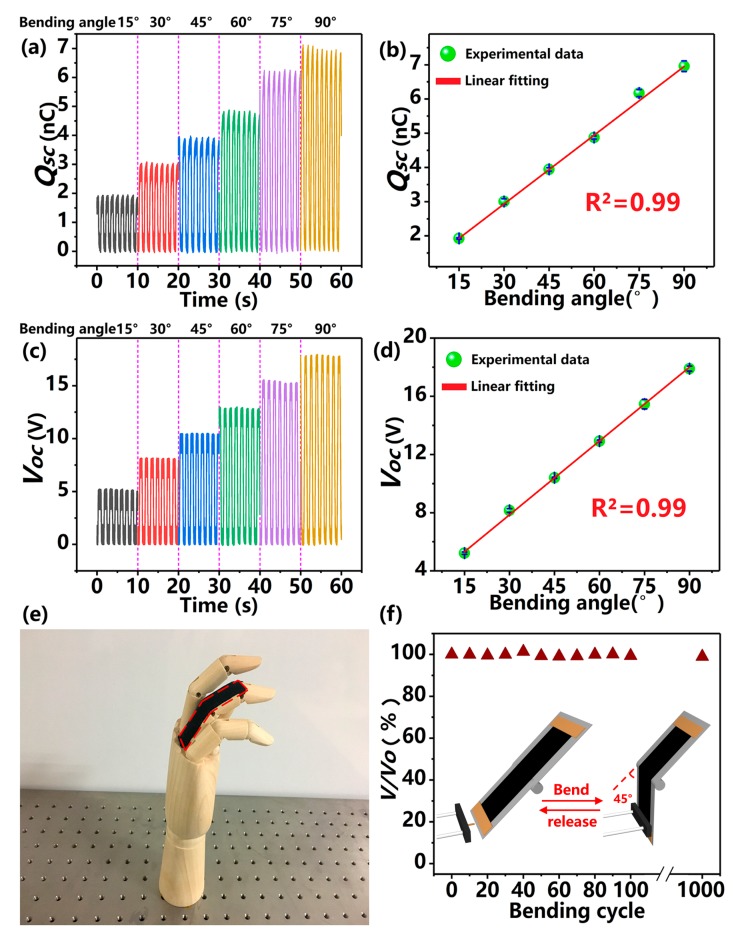
Dependence of the electrical outputs on the bending angle. (**a**) The measured *Q_SC_* of the KT-TENG with different bending angles. (**b**) The relationship between *Q_SC_* and the bending angle. (**c**) The measured *V_OC_* of the KT-TENG with different bending angles. (**d**) The relationship between *V_OC_* and the bending angle. (**e**) Photograph of the KT-TENG attached onto an artificial finger. (**f**) The output stability of the KT-TENG operation at a bending angle of 45° with 1000 bending-releasing cycles. The inset shows the experimental setup for the electric output measurement under bending motion.

To verify the above operation mechanism, the KT-TENG is attached onto a bendable hinge with a bending radius of about 1.5 cm to investigate the relationship between electric output signals (*Q_SC_* and *V_OC_*) and bending angle. The bending angle is controlled by the linear motor. The experimental setup for the bending motion is shown in the inset of Figure 5f. The KT-TENG is attached onto a bendable hinge with a bending radius of about 1.5 cm. One end of the hinge is fixed on a bracket while the other end is connected to a liner motor with a rubber rod. With the above setup, the bending angle of the hinge can be controlled by the displacement of the linear motor. When the linear motor moves forward, it will push the hinge to a specific bending angle. While the linear motor moves backward, the hinge will be pulled back and return to its original position. As can be observed in Figure 5a, when the device bends along with the hinge with an increasing step of 15°, the *Q_SC_* increases from 1.9 nC (at 15°) to 3.0 (at 30°), 3.9 (at 45°), and 4.9 nC (at 60°). The change trend can be fitted by a good linear relationship (*R*^2^ = 0.99), as shown in Figure 5b, resulting from the separated surface area being linearly correlated to the bending angles. The values of *V_OC_* are recorded to be 5.2, 8.2, 10.4, and 12.9 V, corresponding to the bending angles of 15°, 30°, 45°, and 60°, respectively (Figure 5c). Figure 5d illustrates that the change trend of *V_OC_* also exhibits an excellent linear behavior. Therefore, if the KT-TENG is attached onto the joint (such as the artificial finger in Figure 5e), the output voltage can directly reflect the bending condition in real-time, which is crucial for biomedical monitoring, prosthesis development, and so on. Furthermore, we also test the stability and durability of the KT-TENG. As can be seen in Figure 5f, when the devices are operated continuously for 1000 bending-releasing cycles, the output voltage shows little degradation, indicating that the KT-TENG is stable for long-term service.

**Figure 6:**
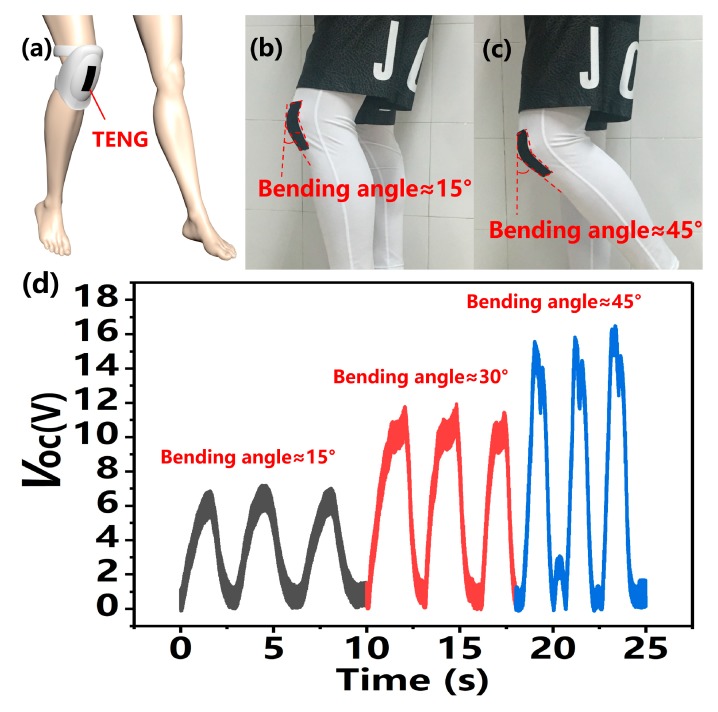

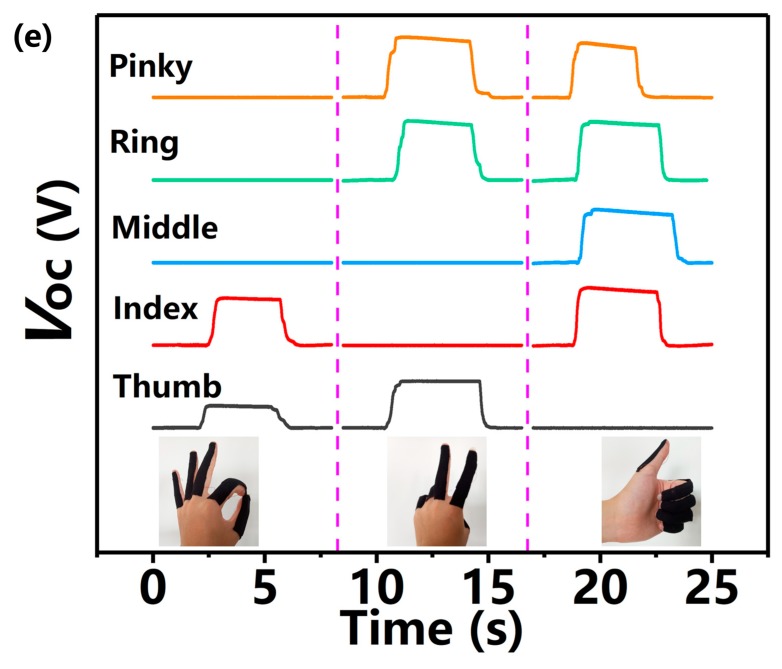
Applications of the KT-TENG. (**a**–**c**) The KT-TENG is attached onto the human knee to monitor the joint motion in real-time. (**d**) The real-time measurement of the *V_OC_* when the device is bent along with the knee at 15°, 30°, and 45°. (**e**) Five KT-TENGs attached onto the human fingers to monitor the finger motion and different gestures in real-time.

To further demonstrate the practical application of the KT-TENG, the device is attached onto the human knee to monitoring the joint motion, as shown in Figure 6a. It is known that the knee joint motion is crucial for human movement, like walking, jumping, etc. Monitoring the bending angle of the knee joint can acquire a great deal of useful information for sports, entertainment, and healthcare. Figure 6b,c depict the KT-TENG bending with the knee joint from 15° to 45°. As shown in Figure 6d, the recorded *V_OC_* can directly distinguish the bending angles of the knee, verifying the potential in a self-powered active human motion sensor. Moreover, the KT-TENG can be further applied for human gesture detection, which is important for sports, entertainment, and healthcare [20,41]. Since the kinesio tapes are of superior flexibility, like human skin, and of good biocompatibility without skin irritation, the KT-TENG can be directly attached to human fingers. After installing five KT-TENGs to the fingers, the self-powered sensors can monitor the motion of each finger and different gestures in real-time, such as the “OK” sign, “V” sign, and “thumbs-up” sign (as shown in Figure 6e), which may be practically applied in a smart glove, prosthetic hand, and human-machine interfacing. 

## 4. Conclusions

In summary, we have demonstrated a stretchable, flexible, and wearable TENG based on kinesio tapes as a self-powered active human motion sensor. The TENG consists of two triboelectric layers (PET and Kapton films) with a back electrode and two kinesio tapes as flexible and stretchable substrates, which has simple fabrication and a compact structure. When the TENG is stretched with certain displacements, an electric output signal will be generated between the two electrodes. Both *Q_SC_* and *V_OC_* will linearly increase with the displacements and bending angles. Moreover, the TENG can be applied as a wearable sensor to monitor the movement of the human knee joint and human gestures in real-time. This work provides a new design and approach for self-powered active human motion sensor which may promote the rapid development of this field.
